# Using social media to crowdsource collection of urine samples during a national pandemic

**DOI:** 10.1007/s11255-022-03108-5

**Published:** 2022-01-26

**Authors:** Elijah P. Ward, Sarah N. Bartolone, Prasun Sharma, Michael B. Chancellor, Laura E. Lamb

**Affiliations:** 1 Beaumont Health, Royal Oak, MI USA; 2 Oakland University William Beaumont School of Medicine, Rochester, MI USA

## Abstract

The COVID-19 pandemic and subsequent lockdown had a substantial impact on normal research operations. Researchers needed to adapt their methods to engage at-home participants. One method is crowdsourcing, in which researchers use social media to recruit participants, gather data, and collect samples. We utilized this method to develop a diagnostic test for Interstitial Cystitis/Bladder Pain Syndrome (IC/BPS). Participants were recruited via posts on popular social-media platforms, and enrolled via a website. Participants received and returned a mail kit containing bladder symptom surveys and a urine sample cup containing room-temperature preservative. Using this method, we collected 1254 IC/BPS and control samples in 3 months from all 50 United States. Our data demonstrate that crowdsourcing is a viable alternative to traditional research, with the ability to reach a broad patient population rapidly. Crowdsourcing is a powerful tool for at-home participation in research, particularly during the lockdown caused by the COVID-19 pandemic.

## Introduction

The COVID-19 pandemic has massively impacted everyday life, shutting down nonessential services and forcing people to shelter in place for weeks at a time. One of the many effects of the global lockdown was the decrease in clinical research with the closing of research labs. With non-COVID-19 research activities suspended through a significant portion of 2020, nonessential researchers and scientists had no laboratory access [1]. A survey of 881 life scientists revealed that 57% reported some loss of work, stemming from decreased laboratory access [2]. This represents a financial problem, as costly experiments, such as those involving animal models, may need to be repeated. In a survey of postdoctoral fellows, 80% reported difficulty in performing experiments and discussing ideas with colleagues [3]. The National Institute of Health’s survey of principal investigators and research leaders revealed extreme concerns with institutional financial stability and productivity of research [4]. Recruiting for a clinical trial during the pandemic also represents an ethical dilemma, as researchers and study participants could be potentially exposed to SARS-CoV-2 during study visits. Because traditional research recruitment and participation methods were hampered by the COVID-19 pandemic, different approaches were necessary. Crowdsourcing, where researchers employ modern communication methods to recruit participants and gather data, is one such approach.

Crowdsourcing as a research method has gained traction in the medical community. Increasing ease of communication and data collection via the internet and social media has resulted in innovations in public health, education, communication, and ethics [5, 6]. By utilizing social media, researchers are able to advertise their studies, recruit and engage with participants, and gather data. Mail-based collection of samples allows for participation from the safety of home. It also has the potential to significantly lower costs while increasing sample size, compared to traditional research [7]. Crowdsourcing also has the benefit of improved study completion. We believe that these benefits make crowdsourcing an ideal tool for reaching groups suffering from rare diseases that are poorly understood or restrict travel, particularly during the COVID-19 pandemic, when participants were not able to leave their homes. Interstitial Cystitis/Bladder Pain Syndrome (IC/BPS) is one such disease that benefits greatly from an at-home, social media-based crowdsourcing approach.

IC/BPS is a debilitating chronic disease that affects roughly 3–8 million women and 1–4 million men in the United States [8, 9]. Approximately 10% of IC/BPS cases present with Hunner’s lesions, which are visible via hydrodystension and cystoscopy; however, the majority of those suffering with IC/BPS do not present with lesions [10]. Diagnosis of IC/BPS is based on patient reported symptoms and exclusion of other diseases with overlapping clinical presentation. Therefore, a non-invasive, rapid, diagnostic test is required. We sought to develop a urine-based biomarker test by crowdsourcing urine from both IC/BPS patients and asymptomatic control participants, utilizing social media and other web-based recruitment tools to reach a large population. We then sought to determine the usefulness and efficacy of these recruitment methods during a nation-wide lockdown and pandemic.

## Methods

### Study recruitment and enrollment

All study materials were approved by the Beaumont Health Institutional Review Board (IRB# 2019–266). A study website was created using Wix, a site development tool. This website contained study background videos, a link to the enrollment survey, shipping information videos, and frequently asked questions. A form for contacting the research team via email was also included. The enrollment survey was created using SurveyMonkey, a Health Insurance Portability and Accountability Act (HIPAA)-compliant survey website. The enrollment survey included demographic information, health history, and IC/BPS diagnosis information. Participants were asked to provide a name and address to which we sent a collection kit.

We partnered with the Interstitial Cystitis Association (ICA), an IC/BPS patient advocacy group, to reach a large audience of people suffering from IC/BPS. The patient advocacy group sent out emails informing members of the study, with links to the recruitment website. The study was also advertised via Google Ads, appearing under the search terms “IC”, “IC/BPS”, “interstitial cystitis”, and “urology study”. Social media posts on websites and phone software applications Instagram, Facebook, and TikTok were used to advertise the study and recruit participants. Once a potential IC/BPS participant had completed the enrollment survey and met enrollment criteria, and consented to participate, an at-home collection kit was prepared and shipped to them. IC/BPS participants were asked to find a male and female of similar age with no bladder health history willing to participate as control samples; inability to find control participants did not exclude the person with IC from participating in the study. Control participants who discovered the study independently were allowed to enroll via the study website.

### At-home collection kit and mailing

At-home collection kits were sent to the address provided by the study participant via FedEx. The mailing kits contained 3 boxes for the IC/BPS participant, male control, and female control. Each box was a self-contained independent sample return mailing kit. All three boxes contained study information sheets, bladder symptom surveys, a urine collection cup containing a room-temperature urine preservative (Norgen Biotek, Thorold, Ontario, Canada), and a pre-paid United States Postal Service (USPS) return mailing envelope. The urine preservative stabilizes proteins and nucleic acids in samples and allows them to be shipped at ambient temperatures, while also inactivating bacteria and viruses in the sample. The male and female control boxes also included demographic survey for the control participants, mimicking the information collected from IC participants during their enrollment survey. All return boxes contained a non-reversible temperature recording label, which indicated how long the package was exposed to temperatures exceeding 30º C. At this temperature, the preservative no longer protects the biomarker proteins of interest from degradation. Bladder symptom surveys included the Interstitial Cystitis Symptom/Problem Index (ICSI/PI), the Overactive Bladder questionnaire (OABq), the Pelvic Pain, Urgency, and Frequency questionnaire (PUF), and the Visual Analog Scale (VAS).

### Urine sample collection and return shipping

Participants were asked to provide a urine sample via midstream catch and place it in the biohazard bag containing absorbent material. They were then asked to fill out the included surveys, place the sample and survey in a pre-labeled USPS envelope, and place the envelope in their mailbox.

Instructions for collecting and returning the urine samples were provided in the kit. The paper instructions included directions to the study website, where videos showed how to collect and ship the samples. The study website also contained a frequently-asked-questions section, which provided answers to common questions, and a “Contact Us” form for questions or concerns about the study.

## Results

### Study enrollment goal was met three months after launch

**Fig. 1:**
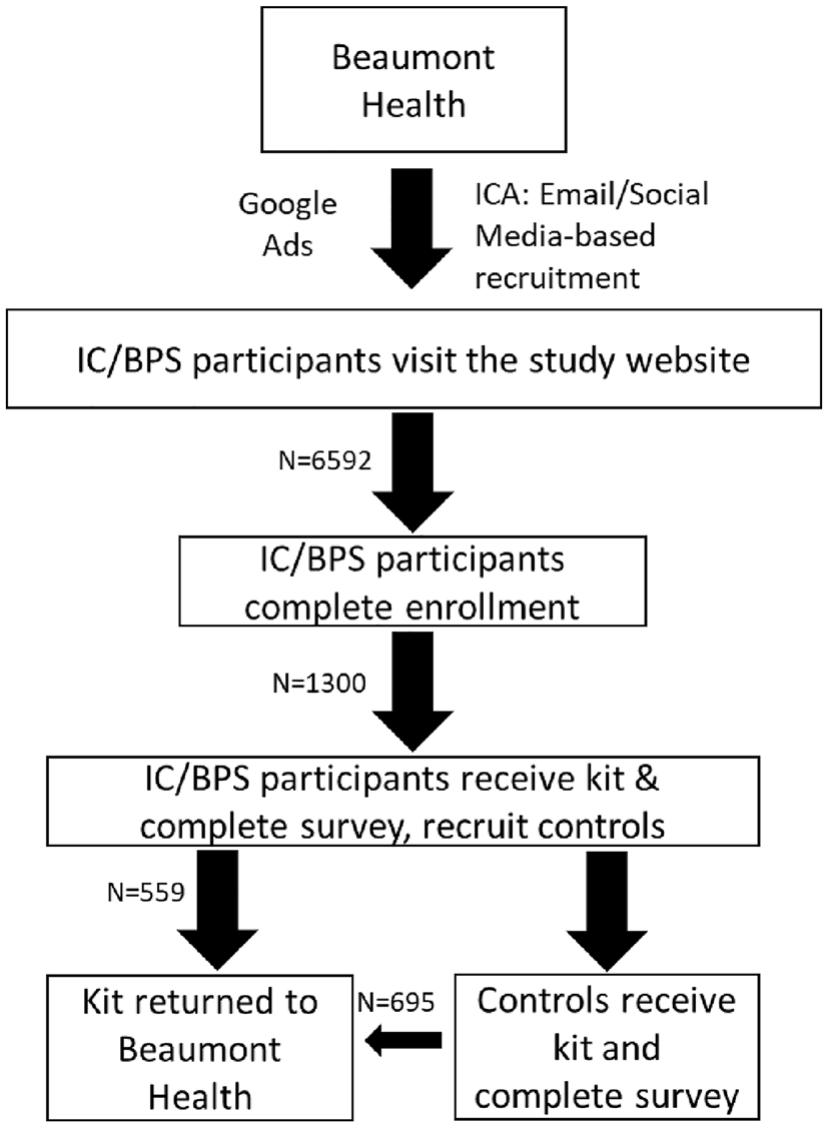
Xxxx

**Fig. 2:**
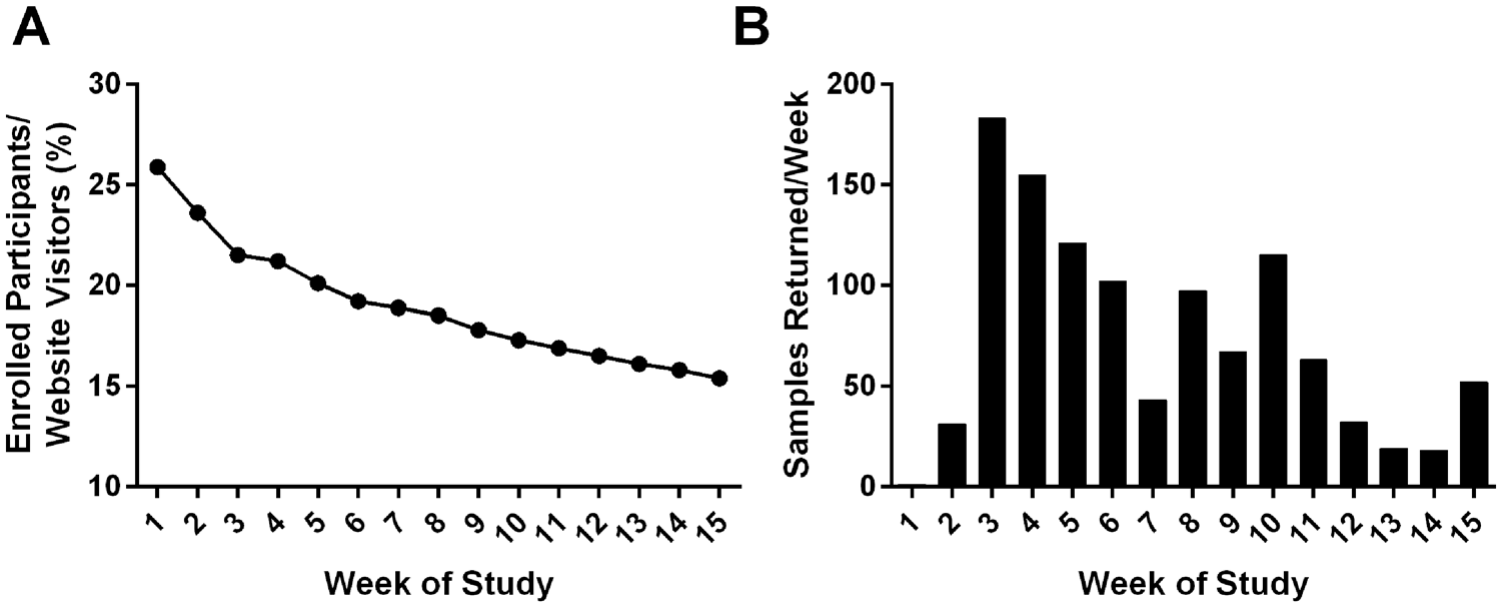
Yyyy

The study was launched September 14, 2020 and closed December 26, 2020. The study recruited 1,254 IC/BPS and control samples from all 50 United States (Fig. 1). The first week of study enrollment had the highest percentage of enrolled participants versus unique website visitors (for the purposes of this study, a “unique website visitor” is only counted once for website visitation, regardless of return visits); this percentage slowly decreased through the duration of the study (Fig. 2A). Sample return rate was highest at week 3 of the study. There was a 2 week average turnaround time from initial enrollment to sample return. Rate-of-return fell slowly over time but rose slightly at weeks 8 and 10, when the patient advocacy group sent reminder emails to participants to return their kits (Fig. 2B).

### Social media posts using video attracted the most website visitors

**Fig. 3:**
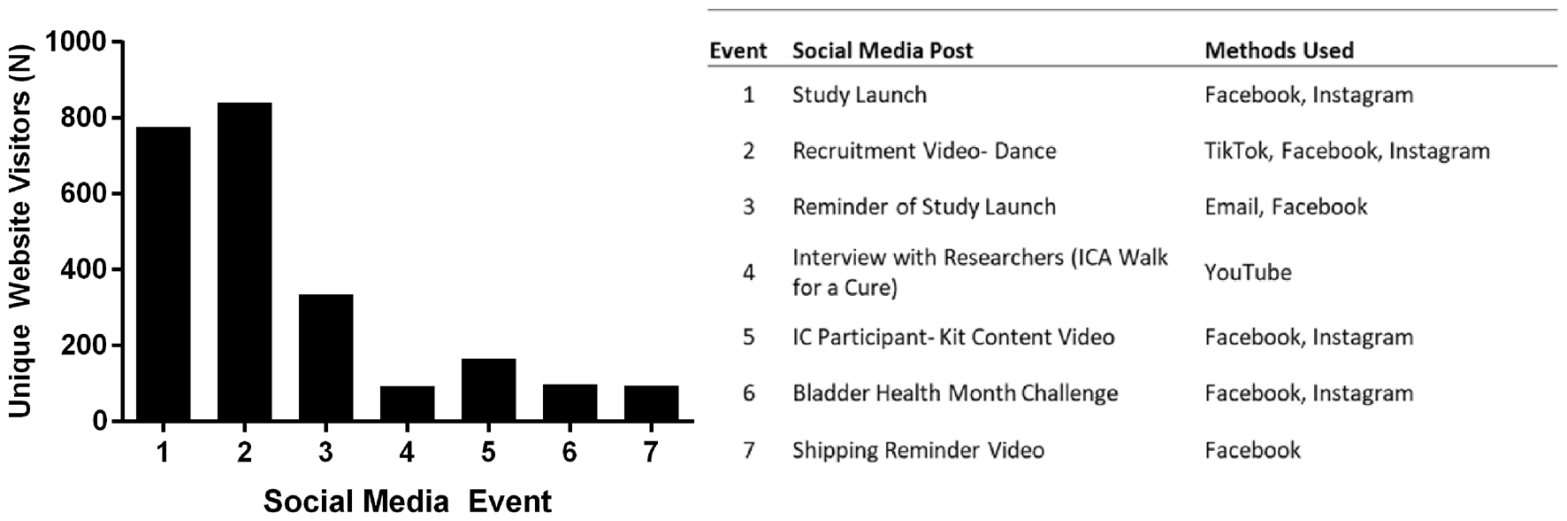
Zzzz

Seven major social-media posts were made during the enrollment period (Fig. 3). The posts that were the most effective were posted on multiple social-media sites. The social-media post with the largest impact on unique website visitors was an 11-s TikTok video advertising the study; this video was also re-posted to Facebook and Instagram.

### Majority of participants used mobile devices to enroll and visit website

**Fig. 4:**
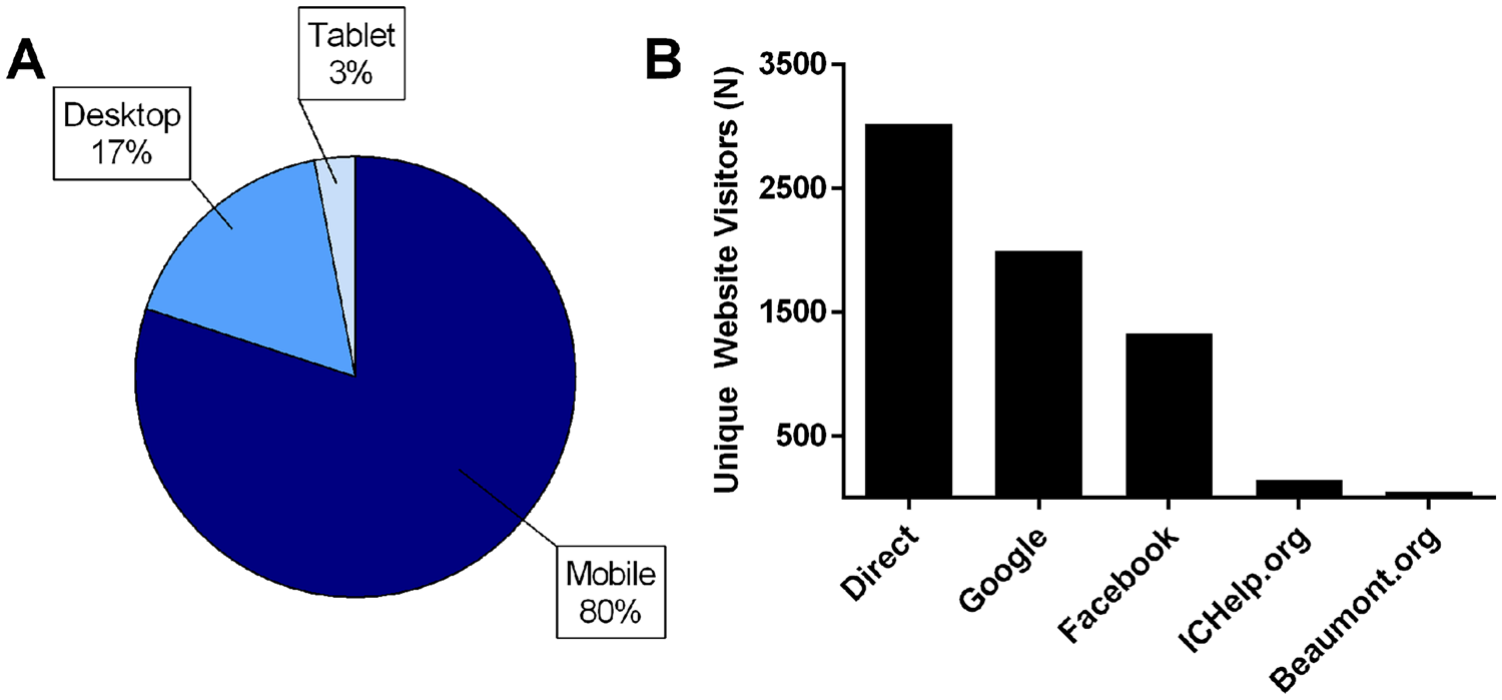
Xxxx

80% of unique website visitors used their mobile devices to visit the study website (Fig. 4A). Mobile devices are responsible for 61% of US website visits, though social networks are predominantly accessed via personal computers [11].

### Google and Facebook are effective tools for study recruitment

Visitors discovered the study website in multiple ways (Fig. 4B). Of these methods, the most common was a direct link, found in recruitment emails. Other methods included Google links (through organic searches or Google Advertisements) and Facebook posts by the ICA and other interested individuals. Finally, the ICA’s website and Beaumont Health’s website resulted in moderate traffic over the study period.

## Discussion

This study confirms the versatility and usefulness of social media-based recruitment and crowdsourcing for clinical sample collection. Social media-based recruitment and crowdsourcing have significant benefits over traditional recruitment methods. This is especially evident as this study was completed during the fall of 2020, in the middle of the COVID-19 pandemic. Social media-based recruitment gathers participants at a significantly faster rate than traditional clinical sample collection. In this study, both clinical sites recruited ten times fewer participants than the at-home, social media-based recruitment in the same three-month period. This may be due to the increased number of people at home and the ease of at-home recruitment and participation during the COVID-19 pandemic lockdown. Online recruitment and crowdsourcing of samples also bypass the geographical limitations of traditional studies; where a normal clinical study may recruit a population in the immediate geographic area around the site(s), a crowdsourcing study with online recruitment methods can recruit participants from all over the country. Another benefit of social-media/web-based recruitment is the ability to tailor to specific target populations. By tailoring recruitment advertisements and partnering with specialized patient advocacy groups, it is possible to target a subset of the normal population, enhancing recruitment goals. Finally, online recruitment and at-home participation is safer than traditional recruitment methods during a pandemic. As SARS-CoV-2 is not highly transmitted on surfaces, it is advantageous to receive a sample kit containing survey responses and biological samples by mail, rather than in-person, where the risk of viral transmission is significantly higher [12].

Online recruitment and crowdsourcing are not without their disadvantages. The biggest disadvantage of crowdsourcing studies is the absence of a patient medical record. For studies such as this that gather both diseased and control samples, researchers are trusting that the participant answers questions honestly, rather than relying on a clinician-confirmed diagnosis. Studies utilizing these methods also frequently lack a personal component. For example, this study had no face-to-face or video-based interaction with participants; while there were email and phone hotline for participant questions and concerns, the lack of personal interaction may have dissuaded some people from participating. In this study, this could not be avoided, as we sought to eliminate participant-physician interaction due to the COVID-19 pandemic. Lack of control participation was also challenging. Control participants who were not recruited by an IC/BPS participant rarely returned their surveys and urine sample. We have observed this in a previous study [13], and tried to address it by including paper surveys for control participants, rather than directing them to a website to complete surveys.

One of the challenges in completing a successful social media-based research study is preparing user-friendly study materials, such as advertisements and websites. In this study, 80% of unique website visitors accessed the study website from their mobile devices. This may be due to users accessing social media more frequently from their phones versus personal computers, as these are app-based platforms. It is also important to have multiple advertisement methods to reach the largest number of potential participants. In this study, we partnered with a patient advocacy group for primary recruitment, created a Google Ad, and optimized the search engine results for the study website. The patient advocacy group advertised the study via direct emails and social-media posts on Facebook, Instagram, and TikTok. The posts that were linked to multiple social-media platforms resulted in the largest spike in unique website visitors. Social media-based recruitment studies can also grow on their own once they are large enough. In this study, a post advertising the study was made on an IC/BPS forum by a user that was not affiliated with our research group nor the patient advocacy group. In another instance, a clinic in South Dakota discovered the study via a social-media post and began to advertise locally for recruitment. We are currently analyzing the large data set from the crowdsource collection via machine learning, a method we recently described [14]. Optimizing the algorithm with big data takes time, but we believe it will provide fruitful results towards developing an IC/BPS diagnostic test.

The final consideration for an online recruitment/crowdsourcing study is the timing of study launch, especially during a national lockdown due to a spreading pandemic. This study was initially prepared for launch in the first quarter of 2020; however, there were multiple problems that we attempted to address, resulting in delay of initial study launch. Firstly, we wanted to ensure that the mail kits and urine samples could not transmit SARS-CoV-2. We waited until publication of research studies that declared surface transmission risk via mail was minimal. We also wanted to confirm that the room-temperature urine preservative prevented transmission of the SARS-CoV-2 virus. The state of the US Postal Service was also considered at the planned time of launch in early 2020. The mail system became backlogged due to the nation-wide lockdown. Finally, full laboratory staff was not allowed in the research facility initially during the pandemic; we were required to stagger our on-site presence, which would have made processing the rapid influx of samples much more difficult. Therefore, the study was postponed until sufficient research staff was available to process incoming samples.

This novel method is potentially limitless in application; though focused on IC/BPS in this study, crowdsourcing and social media-based recruitment could be applied to many urological diseases including overactive bladder, BPH, prostate cancer and bladder cancer, by utilizing existing social-media groups and disease interest groups to spread awareness of the study. While collecting biological specimens can be a challenge, the existence of room-temperature urine preservatives allows researchers to analyze a large volume of samples that are traditionally difficult to acquire due to geographic limitations.

Conclusion: Social media is a powerful untapped resource for researchers. By advertising via social media, it is possible to rapidly gather data from a larger population and geographical region. These new recruitment and participation methods are even more useful during the COVID-19 pandemic, which hampered and sometimes halted normal research operations. Using these methods, we were successful in gathering over 1254 control and IC/BPS urine samples in 3 months from across the country, aiding us in our goal of developing a urine-based diagnostic test for IC/BPS (see Table 1).

**Table 1:** Crowdsource IC/BPS and control demographics

	IC/BPS	Control
No. of participants	559	695
Age (mean ± SD, range)	49.8 ± 16.4 (19–91)	48.0 ± 16.8 (19–95)
No. of males (%)	30 (5%)	401 (58%)
No. of females (%)	529 (95%)	294 (42%)
ICSI/PI (mean ± SD)	23.2 ± 8.1	5.1 ± 5.3
OABq (mean ± SD)	68.9 ± 24.6	25.4 ± 12.1
PUF (mean ± SD)	19.4 ± 6.5	3.3 ± 4.0
VAS (mean ± SD)	5.1 ± 2.3	1.0 ± 0.7

## Data Availability

Data available upon reasonable request from corresponding author.
